# Graduating Nursing Students' Self‐Assessment of Clinical Competence and Need for Further Training: A Nordic Cross‐ Sectional Study

**DOI:** 10.1002/nop2.70364

**Published:** 2025-11-17

**Authors:** Lotta Eronen, Camilla Strandell‐Laine, Sigrid Wangensteen, Auvo Rauhala, Irene Aasen Andersen, Jette Henriksen, Margrét Hrönn Svavarsdóttir, Lisbeth Fagerström

**Affiliations:** ^1^ Åbo Akademi University Vaasa Finland; ^2^ Arcada University of Applied Sciences Helsinki Finland; ^3^ Lovisenberg Diaconal University College Oslo Norway; ^4^ University of Turku Turku Finland; ^5^ Novia University of Applied Sciences Turku Finland; ^6^ Norwegian University of Science and Technology Gjøvik Norway; ^7^ Western Norway University of Applied Sciences Førde Norway; ^8^ Nord Universitet Levanger Norway; ^9^ University of Akureyri Akureyri Iceland

**Keywords:** clinical competence, Nordic countries, nursing curriculum, nursing education, self‐assessed clinical competence

## Abstract

**Aim:**

The study aimed to describe and analyse Nordic nursing students' self‐assessed clinical competence and their perceived need for further training at the end of their bachelor's studies.

**Design:**

A cross‐sectional survey.

**Data Sources:**

From five Nordic countries, and 13 different universities, 291 survey answers were included. The Professional Nurse Self‐Assessment Scale of clinical core competencies (PROFFNurse SAS II) was used for data collection.

**Results:**

Graduating nursing students rated their clinical competence highest in acting ethically, taking responsibility for their decisions, and seeking help, and lowest in assessing patients' health via phone, email, or other health technology solutions, and in professional development. The highest need for further training was reported in medication interactions and side effects and differential diagnosis in health assessment. More than half of the respondents want to work in specialised healthcare after graduation, while less than a quarter want to work in primary care. One in five respondents expressed an intention to leave their career and low scores on self‐assessed clinical competence were associated with increased intentions to leave.

**Conclusion:**

The findings highlight the need for educational institutions to evaluate curricula to enhance graduating nurses' clinical competence in the areas needed, especially within eHealth.

**Implications for the Profession:**

An effective competence development program is necessary to strengthen the competence and career path of newly graduated nurses.

**Impact:**

Educational institutions play a crucial role in developing competency‐based programs that meet quality standards and address current and future health needs, as well as global challenges. By assessing the clinical competence of graduating nursing students regularly nursing education can be developed based on the findings, which promotes high‐quality patient care.

**Reporting Method:**

STROBE guidelines were applied.

**Patient or Public Contribution:**

Graduating nursing students from the Nordic countries participated in the study and contributed to this research by answering the survey.

## 1 Background

Evaluating clinical competence plays a central role in identifying skill gaps, enhancing the quality of nursing education and continuing professional development, and ensuring safe, effective, and high‐quality care (Kajander‐Unkuri et al. 2014, 2021; Gardulf et al. 2016; Lejonqvist et al. 2016). Self‐assessment as an evaluation method of clinical competence enables nurses and students to recognise their strengths and identify areas for improvement, which is essential for promoting evidence‐based practice (Kajander‐Unkuri et al. 2014, 2021; Gardulf et al. 2016; Lejonqvist et al. 2016).

The International Council of Nurses, defines nursing competence as the integration of knowledge, skills, judgement, and attributes required to practice responsibly within a specific role and context (ICN—International Council of Nurses 2023b). Clinical competence has been broadly defined, but in brief as a dynamic and ongoing process that integrates knowledge, skills, attitudes, and critical thinking abilities (Lejonqvist et al. 2016; Fagerström 2021; Lejonqvist 2018; Nabizadeh‐Gharghozar et al. 2021). Clinical competence encompasses not only technical knowledge and skills but also personal values and ethics, shaped by professional and life experiences, which enable nurses to deliver quality care through a person‐centered, ethical approach in direct patient contact (Lejonqvist et al. 2016; Fagerström 2021; Lejonqvist 2018).

Preparing nursing students to engage with global health challenges such as the growing prevalence of chronic diseases, multi‐morbidity, and aging populations further underscores the importance of systematically evaluating clinical competence during nursing education (Holmgren 2017; World Health Organization 2020; State of Health in the EU: Companion Report 2017). Educational institutions play a central role in developing competency‐based programs that adhere to quality standards, address relevant healthcare needs, and adapt to the evolving demands of the healthcare sector (World Health Organization 2020, 2021; Pepito and Locsin 2019; ICN—International Council of Nurses 2023a). Studies show that a well‐educated nursing workforce significantly reduces mortality rates (Aiken et al. 2002, 2017), therefore assessing the clinical competence of nursing students is necessary for the development of nursing education (Nilsson et al. 2019).

Self‐assessment is a valuable tool for evaluating competence (Lejonqvist 2018; Bradley et al. 2022; Høegh‐Larsen et al. 2022; Forsman et al. 2020), especially when used alongside other methods and with an awareness of its limitations. It is essential to recognise the cognitive biases that can affect self‐assessment: novices often overestimate their abilities, while experts tend to underestimate theirs. Furthermore, individuals who lack competence may not possess the skills to accurately identify their own deficiencies.

To ensure a more accurate and balanced evaluation, self‐assessment should be combined with complementary methods, such as formal examinations (Lejonqvist 2018; Bradley et al. 2022; Høegh‐Larsen et al. 2022; Forsman et al. 2020). When integrated with supportive training, for example, mentoring programs and continuous education and other evaluation strategies, self‐assessment becomes a valuable tool for professional growth.

The absence of standardised tools has led to the use of diverse methods and measures for assessment in education and research (Van Horn and Lewallen 2023). Instruments for self‐assessment of nursing competence have been developed for example, the Nurse Professional Competence Scale (NPC) (Gardulf et al. 2016; Charette et al. 2020) and the Nurse Competence Scale (NCS) (Meretoja et al. 2004), but there are limited validated self‐assessment tools specifically designed to measure clinical competence. A need to develop such tools holding a holistic perspective on clinical competence and a patient‐centered approach with a reasonable level of validity and reliability has been proposed to enhance the evaluation of clinical competence (Reljić et al. 2017; Franklin and Melville 2015; Wu et al. 2016).

The Professional Nurse Self‐Assessment Scale of Clinical Core Competencies (PROFFNurse SAS) is designed specifically to evaluate clinical competence. The scale has been applied in various studies across different educational levels and settings (Berthelsen et al. 2023; Leonardsen et al. 2021, 2019; Taylor et al. 2020, 2021; Willman et al. 2020a, 2020b; Blomberg et al. 2019; Wangensteen et al. 2018; Allvin et al. 2020; Finnbakk et al. 2015; Møller et al. 2021). The questionnaire builds on a theoretical model, The Caring Advanced Practice Nursing Model (Fagerström 2021). The underlying model emphasises the equal importance and interconnection of theoretical‐scientific knowledge, practical skills, and wisdom, supporting an evidence‐ and knowledge‐based approach to delivering high‐quality care. The model incorporates a holistic perspective of the patient and care, integrating its core concepts into the questionnaire (Fagerström 2021).

Previous research (Kajander‐Unkuri et al. 2021; Lahtinen et al. 2014; Råholm et al. 2010) has shown that nursing students generally rate their competence as good. However, there are variations in competence and nursing education between European Union countries, despite the implementation of the European Higher Education Area (EHEA). In Europe graduating nursing students rated their self‐assessed professional competence highest in the areas of “value‐based nursing care” and “medical technical care.” However, they rated themselves lowest in the areas of “legislation in nursing and safety planning” and “education and supervision of staff and students” (Nilsson et al. 2019). Additionally, high ratings have also been found (Gardulf et al. 2016; Lachmann and Nilsson 2021) in “patient‐related nursing” and “value‐based nursing,” while low ratings have been found in “Organization and Development”. This need has also been highlighted in another study where nursing students' competence in leadership and organization of nursing was emphasised (World Medical Association 2013).

Studies using the Professional Nurse Self‐Assessment Scale of Clinical Core Competencies II (PROFFNurse SAS II) have revealed that nurses have a significant need for further education in various areas (Allvin et al. 2020; Taylor et al. 2020; Wangensteen et al. 2018) and that the clinical competencies increase significantly during the first year, and the need for further education decreases over time, except for critical thinking (Willman et al. 2020b). Specifically, nurses required more knowledge on the interactions and side effects of different types of medications and in improving their skills in assessing patients' health needs and providing health‐promoting advice and recommendations to patients over the phone (Allvin et al. 2020). High ratings of clinical competence, as assessed using the PROFFNurse SAS II questionnaire, were observed in areas such as multiprofessional collaboration, taking responsibility, and ethical practices (Allvin et al. 2020). Master's level students in advanced practice nursing, as well as postgraduate nurses, rated their ability to take responsibility highly, as well as their proficiency in multiprofessional teamwork and ethical work practices (Taylor et al. 2020; Wangensteen et al. 2018). However, they also indicated the greatest need for further education in medication interactions and effects and the importance of further education on differential diagnoses was emphasised (Taylor et al. 2020; Wangensteen et al. 2018).

Electronic equipment received low scores, despite the perceived need for further training in this area being rated as average (Taylor et al. 2020). The authors (Taylor et al. 2020) concluded that the students in the study might have had limited experience using electronic devices for patient communication, or they may not have had access to such devices in their workplace (Taylor et al. 2020).

Previous clinical work experience and higher education level were not found to have a significant impact on the clinical competence or need for further education (Taylor et al. 2020), however, working in healthcare while studying contributed to higher levels of competence (Gardulf et al. 2016). The educational environment during the final clinical placement has a positive impact on the overall competency of nursing students (Kajander‐Unkuri et al. 2014). High levels of empowerment among nursing students are associated with higher self‐rated levels of generic competence and a reduced intention to leave the profession (Visiers‐Jiménez et al. 2022). It has been pointed out that educators need to consider whether nursing students are adequately equipped to deal with work‐related stressors, as well as being part of creating satisfactory working conditions (Heinen et al. 2013). As previously noted, the initial years following graduation can be a stressful and pivotal time for newly graduated nurses, with significant implications for their future career trajectories (Flinkman and Salanterä 2015; Soerensen et al. 2023; Heinen et al. 2013; Flinkman 2014; Flinkman et al. 2010). Therefore, it is important to examine the current situation concerning clinical competence in terms of potential knowledge gaps and future nursing competencies (Willman et al. 2020a).

**FIGURE 1:**
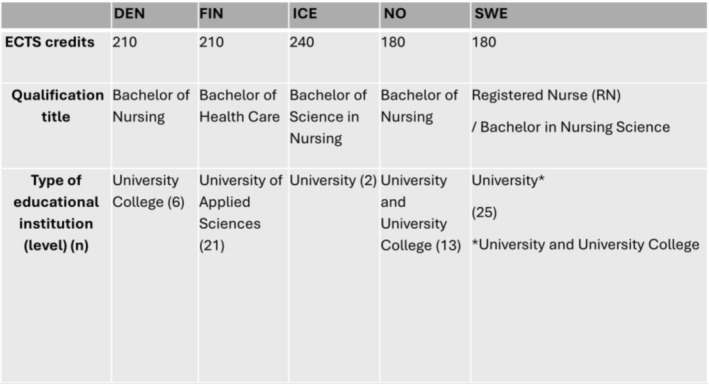
Nursing education in the Nordic countries.

General nursing education in the Nordic countries is structured according to EU directives (2013/55 EU), and should consist of a minimum of three years of study and include at least 4600 h of both theoretical and practical studies (EUR‐Lex 2005, 2013). The theoretical studies must be at least one‐third and the practical studies (clinical practice) at least half of the total hours (2300 h, 90 ECTS). The theoretical studies consist of professional knowledge, skills, and competencies and the practical studies of learning through teamwork, leading, and organising direct patient contact (EUR‐Lex 2005, 2013). The length of the education varies from 180 ECTS to 240 ECTS. Denmark and Finland both require 210 ECTS, while Iceland requires 240 ECTS. Norway and Sweden both require 180 ECTS (Eronen et al. 2023). Clinical practice accounts for 90 ECTS across all countries, though course lengths vary. The bachelor's degree includes 180 credits in Sweden and Norway, representing 50% clinical practice, 210 credits in Denmark and Finland, representing 42.8% clinical practice, and 240 credits in Iceland, representing 37.5% clinical practice (Henriksen et al. 2020; Gunnarsdottir et al. 2021). An analysis of national guidelines in Nordic nursing education showed that the Nordic countries complied well with EU regulations, listing all necessary competency areas in their national nursing education guidelines. However, there were differences in structure and content, leading to a lack of uniformity (Eronen et al. 2023). Nursing education in the Nordic countries is presented in Figure 1.

There are known challenges in fulfilling clinical placements in all necessary areas and in meeting the societal changes in health care related to the competence required of general nurses; therefore, for example, some clinical hours in the Nordic countries are allowed to be replaced by time in a simulation environment (Henriksen et al. 2020). Variations exist regarding the duration of the bachelor's degree programs (Eronen et al. 2023). Research has suggested that nursing education in the Nordic countries should be investigated to determine the quality of education and competency of nursing students upon graduation (Eronen et al. 2023). To investigate the clinical competence of nursing students at the time of graduation, collecting self‐reported data is a useful approach (Gardulf et al. 2016; Fagerström 2021; Lejonqvist 2018).

Evaluating clinical competence from a Nordic perspective can be seen important (Henriksen et al. 2020; Löfmark et al. 2015; Hølge‐Hazelton et al. 2020; Norlén et al. 2024), as the Nordic countries share languages, cultures, and adherence to EU directives, which create unique opportunities and challenges concerning collaboration and the labor market. Consistent competence assessments are essential to maintain high standards of care, as nurses may work across borders within the region. Harmonised evaluations (Lahtinen et al. 2014; Humar and Sansoni 2017; Cabrera and Zabalegui 2021) of clinical competence ensure that all nurses, regardless of their specific country of training, get meaningful education and are prepared to deliver safe and evidence‐based care. This alignment with EU directives supports the goal of providing equitable healthcare services across the Union, safeguarding patient safety, and ensuring quality care throughout the countries (Cabrera and Zabalegui 2021; European Observatory on Health Systems and Policies et al. 2016).

In this study, the aim was to describe and analyse Nordic nursing students' self‐assessed clinical competence and their perceived need for further training at the end of their bachelor's studies.

### 1.1 Research Questions

The research questions of this study are as follows:

1. How do Nordic nursing students assess their clinical competence and their perceived need for further training before graduation in the final semester of their bachelor's studies?
2. Are there any possible associated variables for example, age, gender, intention to leave the career, the primary desired work location, or a possible association between students' self‐assessed clinical competence and their perceived need for further training?

## 2 Methods

### 2.1 Study Design

A cross‐sectional survey design was chosen.

### 2.2 Questionnaire

The Professional Nurse Self‐Assessment Scale of Clinical Core Competencies II (PROFFNurse SAS II) (Taylor et al. 2021) which was used in the present study is a developed and modified version of the previous validated questionnaire PROFFNurse SAS I (Finnbakk et al. 2015). The questionnaire containing 50 items measures both self‐assessed clinical competence and the need for further training of nurses at different educational levels from a holistic perspective and focusing on patient centered care. The questionnaire asks for (a) self‐assessment of clinical competence on a scale of 1 to 10 (A scale), where 1 indicates low and 10 high competence, and (b) need for further training (B scale), where 1 indicates low need and 10 high need for further training for each item (Wangensteen et al. 2018; Taylor et al. 2021). An earlier version of the PROFFNurse SAS questionnaire has been evaluated for content validity and reliability in the Norwegian context (the Cronbach's alpha value for the A‐scale was 0.936) (Finnbakk et al. 2015). However, the questionnaire has been further developed and was named PROFFNurse SAS II. The PROFFNurse SAS II version was subject to translation into the Nordic languages (from Norwegian to Swedish, Finnish‐Swedish, Finnish, Danish, Icelandic, and English) (Anåker et al. 2024). The translation process has been carried out in several stages involving linguistic specialists, proofreading, blinded back translation, comparisons, and adjustments based on expert assessment. Pilot testing was conducted to ensure understanding of the items (Anåker et al. 2024). No revisions of the questionnaire PROFFNurse SAS II were carried out in this study.

### 2.3 Data Collection

Data collection was conducted using an online electronic questionnaire, the ProffNurse SAS II, along with a set of questions on background factors. The study included students enrolled in the final semester of bachelor's programs in general‐level nursing education across the Nordic countries. Each country used a local language version of the questionnaire to ensure linguistic and cultural relevance (Anåker et al. 2024). Students graduating from programs other than general nursing education at the bachelor's level, such as post‐graduate or master's programs, were excluded from the study. All data was gathered during the participants' final semester.

The questionnaire was distributed to participating countries in compliance with their respective regulations and interpretations of the General Data Protection Regulation (GDPR) (Regulation—2016/679—EN—gdpr—EUR‐Lex 2024). In Norway, the electronic questionnaire link was published on a learning platform, while in other countries, it was sent to respondents either via student email, through a designated university contact person, or directly. Students received two email reminders to encourage participation. Data was collected twice a year to account for students graduating at both the end of the year and in the spring across different countries. The collection period ran from spring 2021 to fall 2022, resulting in a total of four data collections.

### 2.4 Data Analyses

The jamovi version 2.3.21 (The Jamovi Project 2022; R Core Team 2021) software was used for data analyses. Data were expressed as frequencies, percentages, mean, SD, range, minimum and maximum, and 95% confidence interval for mean and OR. See scales and categories of background variables in Table 2.

In inferential statistics mean scores for scale A and scale B represented the PROFFNurse SAS II. Background variables that were included in the binary logistic regression were the following: age (in interval scale), gender (as dichotomous) and intention to leave the career (as dichotomous). The primary desired work location of students in three different categories (categorical, One‐way ANOVA) were analysed with the mean score for scale A and scale B. Assumptions of the one‐way ANOVA analysis and binary logistic regression were tested and met and multicollinearity was omitted. Independent samples t‐test was used to compare the mean score for scale A and scale B between gender. Pearson correlation method was used to analyse the association between students answers on the A and B scale. The quantitative variables were assessed as normally distributed and fit for parametric analysis both visually and by conducting normality assumption checks (histograms, Q‐Q plots, skewness, kurtosis and Shapiro–Wilk tests).

### 2.5 Ethical Considerations

Good scientific practices were followed throughout the study (World Medical Association 2013; Ethical Guidelines for Nursing Research in the Nordic Countries 2003; All European Academies 2017) and the ethical principles of the Declaration of Helsinki (World Medical Association 2013) were followed. A research permit was granted from all participating universities, university colleges, or universities of applied sciences according to their policy by members of the research group. The data was collected in collaboration with a Nordic research project that was approved by the Norwegian Centre for Research Data (803827). Ethical review was not pursued for this study, due to no sensitive data being collected, and confidential data was not included in the data received from the project for this study.

The respondents were informed via email in a covering letter of the voluntariness of participation, confidentiality and the right to withdraw participation in the study at any time. Data protection was emphasised (Regulation—2016/679—EN—gdpr—EUR‐Lex 2024). When feasible, students were provided with verbal information about the study, considering the restrictions imposed by the COVID‐19 pandemic. By answering the questionnaire, the respondents have given their consent to participate. Respondents could be assured that participation in or withdrawal from the study would not affect the grading, as they were in the last semester and had completed their studies and participation was via an electronic questionnaire.

The questionnaire's copyright holders are members of the author's research team. Data transfer was facilitated through specific agreements between the principal investigator, the affiliated universities in the Nordic research collaboration, and the participating researchers. No confidential data was included in the inter‐country data transfers. The collected data will be stored digitally in an anonymised format by the principal investigator of the Nordic research collaboration. Personal data provided by informants will be stored separately from the research data and will remain unlinked during the research phases. Neither personal data nor research data will be transferred outside the Nordic research group. All participant data collected for the Nordic research collaboration will be handled confidentially and in compliance with GDPR (Regulation—2016/679—EN—gdpr—EUR‐Lex 2024). Additionally, the data will be archived in accordance with university regulations.

## 3 Results

### 3.1 Sample Size and Power

A total of 2869 nursing students who were in their last semester and about to graduate from general nursing education at bachelor's level in 13 different universities, university colleges, or universities of applied sciences in Denmark, Finland, Iceland, Norway, and Sweden from spring 2021 to fall 2022 were invited to participate. Out of those invited (2869), 839 students responded. Of the 839 respondents, 339 answered only background questions, leaving 500 that answered at least some questions on The Professional Nurse Self‐Assessment Scale of clinical core competencies (PROFFNURSE SAS II), resulting in a response rate of 17.4%. Complete answers without any missing items were 262. Additionally, 29 respondents (10% of all included) had a maximum of four missing answers out of one hundred (4%) and were also included. Thus, totally 291 respondents, with 29,100 individual answers, were included as the final sample. Of these answers only 49, that is, 0.17%, were imputed using the case mean substitution technique as an imputation method (Fox‐Wasylyshyn and El‐Masri 2005). Little's MCAR test was conducted to verify that data was missing completely at random. Table 1 reports the overview of the process from possible invited nursing students to respondents included in the analysis.

**TABLE 1:**
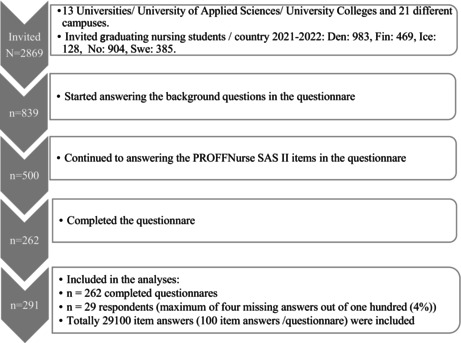
Overview of the process from possible invited nursing students to respondents included in the analysis.

Although the sample was already collected, the sample size calculation was performed to assure that it is sufficient for the planned analyses. The variable used in the analyses was self‐ assessed clinical competence mean, with a SD of 1.2, with the following assumptions: Cohen's *d* effect size = 0.5 (corresponding to a moderate effect and difference in means of 0.6, *α* = 0.01, and power = 0.80). The estimated sample size was 94. Thus, for comparisons of equal‐sized normally distributed groups the required total number of respondents was 188 for two groups. The only country with a sufficient number of participants was Denmark (*n* = 120). Therefore, country‐level comparisons were performed only by estimating the mean and 95% confidence interval.

### 3.2 Characteristics of the Sample

In total, 291 respondents were included in the analysis. The respondents were distributed as follows: Denmark: 120 (41.2%), Finland: 24 (8.2%), Iceland: 53 (18.2%), Norway: 57 (19.6%), and Sweden: 37 (12.7%). The respondents' mean age was 28.7 years (SD: 7.21, range 21–58). The majority, 64%, responded that they primarily want to work in specialised healthcare, 23% in primary care, and 13% in other sectors. Neither gender, nor the primary desired work location of students in three different categories was found to have a statistically significant association with either the mean score for scale A or scale B. Table 2 reports the characteristics of the sample in detail.

**TABLE 2:** Characteristics of sample.

Characteristics	Denmark	Finland	Iceland	Norway	Sweden	Total
Campuses	6	5	2	5	3	
*n* (%)	120 (41.2)	24 (8.2)	53 (18.2)	57 (19.6)	37 (12.7)	291 (100)
Age in years						
Mean	28.5	26.2	29.2	27.8	31.7	28.7
Range	22–55	22–36	22–53	21–49	22–58	21–58
Gender *n* (%)						
Female	111 (93)	21 (88)	51 (96)	52 (91)	31 (84)	266 (91)
Male	9 (7)	3 (12)	2 (4)	5 (9)	6 (16)	25 (9)
Neutral	—	—	—	—	—	—
Where do you primarily want to work after graduation? *n* (%)						
Primary care	26 (22)	5 (21)	6 (11)	22 (39)	9 (24)	68 (23)
Specialised health care	80 (67)	14 (58)	39 (74)	28 (49)	25 (68)	186 (64)
Other	14 (12)	5 (21)	8 (15)	7 (12)	3 (8)	37 (13)
Do you want to change your career choice? *n* (%)						
No	107 (90)	15 (60)	31 (58)	52 (93)	35 (95)	240 (82)
Yes	12 (10)	9 (40)	22 (42)	4 (7)	2 (5)	49 (17)

### 3.3 Highest and Lowest Self‐Assessed Clinical Competence

The highest competence was reported within areas of acting ethically when caring for patients (*M* = 8.69, SD = 1.45), taking full responsibility for one's own actions (*M* = 8.66, SD = 1.60), and being cognizant of when one's own medical knowledge is insufficient when assessing patients' health conditions (*M* = 8.56, SD = 1.63). The lowest competence was reported within areas of assessing patients' health needs by phone, email, or other health technology solutions (*M* = 4.54, SD = 2.68), providing health promotion, advice and recommendations to patients by phone, email or other health technology solutions (*M* = 4.62, SD = 2.70), and improving routines/systems that fail to meet the needs of patients at the workplace (*M* = 5.14, SD = 2.52). Graduating nursing students' top ten highest and lowest self‐assessed clinical competences are reported in Table 3.

**TABLE 3:** Top 10 highest and lowest self‐assessed clinical competence (1–10).

Item highest self‐assessed clinical competence no.	Mean (SD)	Item lowest self‐assessed clinical competence no.	Mean (SD)
24. I act ethically when caring for patients	8.69 (1.45)	45. I assess patients' health needs by phone, email, or other health technology solutions	4.54 (2.68)
32. I take full responsibility for my own actions	8.66 (1.60)	46. I provide health promotion, advice and recommendations to patients by phone, email or other health technology solutions	4.62 (2.70)
39. I am cognizant of when my medical knowledge is insufficient when assessing patients' health conditions	8.56 (1.63)	19. I improve routines/systems that fail to meet the needs of patients at my workplace	5.14 (2.52)
41. I reflect on my actions	8.56 (1.46)	18. I take responsibility for competence development at my workplace	5.21 (2.69)
37. I consult other professional experts when required	8.54 (1.80)	15. I have knowledge of the interactions of various types of medication and what side‐effects they may cause for the patients I am responsible for	5.38 (2.11)
23. I take patients' physical health needs (illness, pain, disabilities, etc.) into account when assessing and planning for the health and life situation of patients	8.39 (1.45)	16. I generate a creative learning environment for staff at my workplace	5.38 (2.52)
34. I understand the consequences my decisions may have for patients	8.35 (1.45)	17. I participate in quality development at my workplace	5.40 (2.55)
40. I document the steps taken in assessing patients' needs for nursing, care and treatment	8.24 (1.55)	8. I interpret, analyse, and reach alternative conclusions about patients' health conditions after a detailed mapping of health history and health assessment (physical examination)	5.98 (1.86)
29. I take an active part in creating a good working environment	8.23 (1.84)	6. I evaluate and modify patients' medical treatment	6.06 (2.06)
30. I put emphasis on patients' own wishes when assessing and planning for nursing care and medical treatment	8.17 (1.50)	44. I have a vision of how nursing should be developed at my workplace	6.25 (2.30)

### 3.4 Highest and Lowest Self‐Assessed Need for Further Training

The highest need for further training was reported in areas of knowledge of the interactions of various types of medications and what side effects they may cause for the patients (*M* = 7.14, SD = 2.49), excluding alternative diagnoses when assessing patients' health conditions (*M* = 6.78, SD = 2.29), and interpreting, analysing, and reaching alternative conclusions about patients' health conditions after a detailed mapping of health history and health assessment (physical examination) (*M* = 6.66, SD = 2.39). The lowest need for further training was reported in areas of taking an active part in creating a good working environment (*M* = 3.79, SD = 2.70), maintaining an ethical approach towards one's own colleagues (*M* = 4.24, SD = 2.74), and consulting other professional experts when required (*M* = 4.40, SD = 2.91). Graduating nursing students' top ten highest and lowest mean scores for the need for further training are reported in Table 4.

**TABLE 4:** Top 10 highest and lowest need for further knowledge/training (1–10).

Item highest need for further knowledge/training no.	Mean (SD)	Item lowest need for further knowledge/training no.	Mean (SD)
15. I have knowledge of the interactions of various types of medication and what side‐effects they may cause for the patients I am responsible for	7.14 (2.49)	29. I take an active part in creating a good working environment	3.79 (2.70)
7. I exclude alternative diagnoses when assessing patients' health conditions	6.78 (2.29)	28. I maintain an ethical approach towards my colleagues	4.24 (2.74)
8. I interpret, analyse, and reach alternative conclusions about patients' health conditions after a detailed mapping of health history and health assessment (physical examination)	6.66 (2.39)	37. I consult other professional experts when required	4.40 (2.91)
1. I am independently responsible for health assessments (systematic physical examination), examinations, and treatment of patients with complicated medical conditions	6.61 (2.27)	32. I take full responsibility for my own actions	4.42 (3.00)
6. I evaluate and modify patients' medical treatment	6.61 (2.49)	24. I act ethically when caring for patients	4.51 (2.96)
11. I have knowledge of the medication effects and treatment for the patients I am responsible for	6.57 (2.73)	30. I put an emphasis on patients' own wishes when assessing and planning for nursing care and medical treatment	4.51 (2.75)
45. I assess patients' health needs by phone, email, or other health technology solutions	6.34 (2.74)	41. I reflect on my actions	4.52 (2.92)
46. I provide health promotion, advice, and recommendations to patients by phone, email, or other health technology solutions	6.26 (2.79)	40. I document the steps taken in assessing patients' needs for nursing, care, and treatment	4.56 (2.80)
18. I take responsibility for competence development at my workplace	6.10 (2.61)	38. I cooperate actively with other health professionals when coordinating patients' nursing, care, and treatment	4.64 (2.81)
19. I improve routines/systems that fail to meet the needs of patients at my workplace	5.96 (2.59)	34. I understand the consequences my decisions may have for patients	4.70 (2.97)

### 3.5 Comparison of Mean Scores Between Nordic Countries

The mean A scale value was higher for Finland and Iceland compared to Denmark, without any overlapping in 95% confidence intervals. The corresponding Cohen's *d* effect sizes of the differences in these two comparisons were +0.67 and +0.63, that is, of a medium effect size. Mean scores per country and their distribution parameters are reported in Table 5.

**TABLE 5:** Mean scale A (self‐assessed clinical competence 1–10) and scale B (need for further knowledge/training 1–10) per country.

Country (ECTS)	Mean (SD)	Mean (SD)	95% CI	Range
	Scale A	Scale B		
Denmark (210)	6.89 (1.12)	5.59 (2.14)	6.69–7.10 (A)	3.02–9.30 (A)
			5.20–5.97 (B)	1.20–10.0 (B)
Finland (210)	7.65 (1.19)	5.20 (2.11)	7.15–8.16 (A)	5.40–9.86 (A)
			4.31–6.10 (B)	2.28–9.88 (B)
Iceland (240)	7.61 (1.04)	4.98 (2.18)	7.32–7.90 (A)	4.98–9.78 (A)
			4.38–5.59 (B)	1.12–9.72 (B)
Norway (180)	7.10 (1.25)	5.52 (2.10)	6.77–7.44 (A)	4.44–9.40 (A)
			4.96–6.08 (B)	1.94–10.0 (B)
Sweden (180)	7.16 (1.13)	5.46 (1.82)	6.78–7.54 (A)	4.64–9.84 (A)
			4.85–6.07 (B)	1.96–8.88 (B)
Total	7.16 (1.17)	5.42 (2.10)	7.03–7.30 (A)	3.02–9.86 (A)
			5.17–5.66 (B)	1.12–10.0 (B)

Abbreviation: ECTS = European Credit Transfer System, indicates the required workload to complete a study programme.

### 3.6 Correlation Between A (Self‐Assessed Clinical Competence) and B (Self‐Assessed Need for Further Training) Questions

Pearson's correlation was performed both (1) between each respondent's each item A and corresponding item B and (2) between each respondent's mean A and mean B. Both correlations proved to be the same, *r* = −0.470, *p* < 0.001. The coefficient of determination *R* ^2^ of this covariation is the square of the correlation, that is, 0.221, or 22.1%.

### 3.7 Intention to Leave the Career

Exploratory analyses were conducted to describe the intentions to leave the career between the Nordic countries. For example, in Finland and Iceland more than a third wanted to change careers, while in other countries, less than a tenth did. Further, a binary logistic regression was performed to assess the effects of age, country, and mean scores for scale A and scale B on the likelihood of intentions to leave the career (with categories ‘Yes’ and ‘No’, the latter of which was the reference category). When assessing the effects of country on an intention to leave the career, a statistically significant difference was found between Finland and Denmark (OR = 7.09, 95% CI = 2.33–21.59, *p* < 0.001) and Iceland and Denmark (OR = 10.88, 95% CI = 4.33–27.37, *p* < 0.001). Additionally, increasing age was found to be associated with decreased intentions to leave the career (OR = 0.90, *p* = 0.014, for each additional year) and according to the marginal means table the probability decreased from 24% to 7% in the age range 22–36 years. Low scores on self‐assessed clinical competence were associated with increased intentions to leave the career (OR = 0.64 for each additional point, *p* = 0.013) and according to the marginal means table the probability increased from 8% to 21%, when the mean score decreased from 8.3 to 6.0 on the A scale. Overall, the model explained 28% (Nagelkerke *R* ^2^) of the variation in intentions to leave the career and the overall model test *p* < 0.001.

## 4 Discussion

The aim of this study was to evaluate the self‐assessed clinical competence and the perceived need for further training of general level nursing students at the end of their bachelor studies, and factors relating to it. The background variables that were included were the following: age, gender, intention to leave the career, and the primary desired work location.

The highest scores for self‐assessed clinical competence were found in the areas of ethical acting, decision‐making, and seeking help. The lowest scores were found in assessing patient health using health technology solutions and professional development. The greatest need for further training was reported in pharmacological skills and differential diagnosis in health assessment. After graduation, most respondents expressed a preference for working in specialised healthcare, while less than a quarter preferred primary care. Additionally, low scores on self‐assessed clinical competence were associated with increased intentions to leave the nursing profession.

To provide quality care, nurses need a variety of competencies. A nurse who has completed general nursing education is prepared and authorised to participate as a member of the healthcare team (ICN—International Council of Nurses 2023b). It is important to note that nurses have different focuses and responsibilities compared to other professions (ICN—International Council of Nurses 2023b; American Nurses Association 2021), such as physicians, although there are legal limits to their professional autonomy. Nursing aims to protect, promote, and optimise health and human functioning. This includes preventing illness and injury, facilitating healing, and relieving suffering through compassionate care. Additionally, nursing involves diagnosing and treating human responses (American Nurses Association 2021). In healthcare, nurses work together with other healthcare and public service professionals to plan, implement, and evaluate care (American Nurses Association 2021).

The need for further training on medication interactions and side effects is consistent with previous studies among post‐graduate and master's level students (Taylor et al. 2020, 2021; Wangensteen et al. 2018). It appears that this need for training persists among nurses as they progress in their careers (Taylor et al. 2020, 2021; Wangensteen et al. 2018). Nurses must possess strong pharmacological skills to prepare and administer medications effectively, as well as to monitor patients' well‐being during and after drug treatment (ICN—International Council of Nurses 2023b; American Nurses Association 2021). Strengthening nursing students' clinical competence in understanding medication interactions and side effects is essential and calls for attention. Enhancing pharmacological skills requires a comprehensive review and revision of nursing education curricula at the bachelor's level. Additionally, clinical supervisors should prioritise this aspect during students' clinical placements (Kajander‐Unkuri et al. 2021). Orientation programs for newly graduated nurses should also be thoughtfully designed, incorporating insights from recent research (Holmgren 2017). Collaboration among educational institutions, clinical teachers, and healthcare organisations is vital to address these gaps effectively (Holmgren 2017; World Health Organization 2020; Pepito and Locsin 2019). Encouraging students to recognise their limitations is equally important, as it can highlight gaps and the need for improvement. To ensure a seamless transition from student to professional nurse, and to promote retention within the profession, career development initiatives must take these needs seriously (Flinkman and Salanterä 2015; Soerensen et al. 2023).

Previous studies have found low scores in organisation and development (Gardulf et al. 2016; Lachmann and Nilsson 2021), in addition to pharmacological competence. The results of this study are consistent with those of previous studies (Gardulf et al. 2016; Lachmann and Nilsson 2021), as the students reported low scores for self‐assessed clinical competence in professional development and quality development in the workplace. This result is somewhat related to the timing of the survey, which was conducted during the initial stages of nursing students' careers. It is, however, important to consider how nursing students and newly graduated nurses with valuable knowledge and competencies can make a greater contribution to the development work at clinical placements and in healthcare organisations. For instance, research has shown (Kajander‐Unkuri et al. 2014) that creating a pedagogical atmosphere during the final clinical placement can positively correlate with overall levels of competence. It is therefore crucial to explore how nurses can enhance their professional development starting from the point of graduation (Kaldal et al. 2024; Koskinen et al. 2023).

Low scores were also reported for clinical competence in providing health promotion, advice, and recommendations through telephone, email, or other health technology solutions. However, eHealth competencies are rated low, yet the perceived need for further training in this area is not ranked highly, which aligns with findings from a previous study (Finnbakk et al. 2015). This raises the question of whether nursing students believe technical eHealth competencies should be learned in the workplace, or if they rarely use these skills and therefore do not prioritise additional training. It seems that the need for further training in pharmacological competence is highly valued based on findings from this study and previous studies (Allvin et al. 2020; Taylor et al. 2020; Wangensteen et al. 2018), while clinical competence in health assessment via digital solutions is equally crucial for patient safety and should not be ignored. It is possible that nursing education is facing challenges in keeping pace with the rapid technological advancements in the healthcare sector (Pepito and Locsin 2019; Gunnarsdottir et al. 2021; Zander 2016). Educational institutions must equip nursing students with the competencies required to meet todays and the future (Holmgren 2017; Pepito and Locsin 2019; Wakefield et al. 2021) needs of e‐health, patient assessment, and the digital evolution of health systems. Health technology and artificial intelligence are playing an increasingly important role in the healthcare setting today and in the future (World Health Organization 2020; Pepito and Locsin 2019). Leading to a need for strong cooperation between nursing education, healthcare organisations, and businesses for the development and implementation of sustainable and ethical health technologies, including artificial intelligence. Interdisciplinary cooperation can be seen necessary to create technologies that meet the needs of both patients and healthcare professionals. As in this study, nurses score high in the areas characterising the core of their profession, in ethical acting and decision‐making. This aspects highlights the importance of involving nurses in the development processes, leveraging their expertise in ethics and person‐centered care to ensure the development of holistic, high‐quality, sustainable, and patient‐safe technological solutions for healthcare (Pepito and Locsin 2019).

The healthcare sector faces challenges from an aging population, a rise in chronic diseases, and multi‐morbidity. Nurse‐led models of care and advanced practice roles are becoming more important as solutions to address these challenges (World Health Organization 2020; State of Health in the EU: Companion Report 2017; Taylor et al. 2020; De Raeve et al. 2024). Clinical competencies in Health assessments and differential diagnosis play a crucial role in the independent decision‐making process of nurses in nurse‐led models, advanced practice roles (World Health Organization 2020; State of Health in the EU: Companion Report 2017; Taylor et al. 2020; De Raeve et al. 2024), and remote consultations (Fagerström 2021; Taylor et al. 2021; De Raeve et al. 2024). Low scores for direct clinical practice regarding patient and health assessments in this study may be related to the early stage of the career, as the population consists of graduating nursing students from general level education, but still requires attention. Health assessment competencies are fundamental for practicing nursing (ICN—International Council of Nurses 2023b; American Nurses Association 2021), and the knowledge gap within this area needs to be further explored.

Graduating nursing students score high in several valuable areas, including acting ethically, responsibility‐taking, reflective decision‐making, teamwork, and self‐awareness. Among these areas, acting ethically when caring for patients was rated highest, while taking an active role in creating a positive work environment was rated the lowest in terms of need for further training. Interprofessional teamwork is emphasised as essential for enhancing care and decreasing hospitalisation rates, an area that received high scores (World Health Organization 2020; State of Health in the EU: Companion Report 2017). When looking at the somewhat intertwining definitions of nursing competence and clinical competence, nursing competence could be said to encompass both clinical expertise and the broader professional role. While clinical competence is a crucial aspect of nursing competence, it does not cover the wider professional behaviours and roles beyond direct patient care, such as interpersonal, and organisational duties involved in nursing practice but is more closely applied to the direct patient contact and clinical setting. Whether these two definitions should be separated has been discussed for a long time and the discussion is ongoing (Nabizadeh‐Gharghozar et al. 2021; Axley 2008; Smith 2012; Notarnicola et al. 2016).

The advanced clinical skills of a nurse can be defined as a combination of theoretical‐scientific knowledge, practical skills, and practical wisdom, which are all evident in their work (Fagerström 2021). In evidence‐ and knowledge‐based nursing, it is important that these different types of knowledge are given equal importance, and a dialogue between the different perspectives is necessary to provide high‐quality care (Lejonqvist et al. 2016; Fagerström 2021; Lejonqvist 2018). The results above indicate that nursing students who graduate possess a solid understanding of ethical decision‐making, a holistic view of human beings, and strong collaboration and consultation skills. It is noteworthy that theoretical perspectives, such as the person‐centered ethical approach (Fagerström 2019, 2021), which form the foundation of the caring science tradition and high‐quality care, are highly valued by graduating nursing students.

Nordic nursing graduates mainly aspire to work in specialised healthcare. More than half of the respondents expressed their interest in working in specialised healthcare. Only one‐fifth of students report an interest in working within primary care, while only one‐tenth express interest in working in other fields. Due to the aging population (State of Health in the EU: Companion Report 2017; World Health Organization 2021; Norlén et al. 2024), there is an urgent need to retain and inspire nurses to work in primary care and geriatrics. This must be considered when developing education, continuous learning, and training programs.

Several studies conducted in Europe have shown similarities in both the highest and lowest levels of self‐assessed clinical competence among graduating nursing students, which is consistent with the results of this study (Gardulf et al. 2016; Nilsson et al. 2019; Lachmann and Nilsson 2021). But also clear differences between countries have been noted in the European Union (EU) (Kajander‐Unkuri et al. 2021; Henriksen et al. 2020; Jokiniemi et al. 2021). In this study, the estimates and their 95% confidence intervals of the self‐assessed clinical competence (A scale) for graduating nursing students did not at all overlap each other's between Denmark and Iceland, and between Denmark and Finland. Even the effect sizes of those differences were of moderate level. However, considering the insufficient power of our study for testing the statistical significance of country‐specific differences, further studies are needed if one is to compare clinical competence and perceived need for further training between countries. Despite variations in the duration of the programs for general nursing education and general nursing curricula in Europe and the Nordic countries, this study does not provide sufficient evidence to determine whether the program's duration affects self‐assessed clinical competence or to compare countries. Thus, in order to meet the high demand for nurses in the future, a harmonisation (World Health Organization 2020; Eronen et al. 2023; Henriksen et al. 2020) of nursing education within the Nordic countries and Europe could be considered, for example, to ensure rapid qualification, a free labor market and standardised care. In the Nordic countries, which share similar languages, there has been considerable mobility for decades that could benefit from harmonisation. Future nursing competencies and areas that need strengthening can still be considered similar across Europe and the Nordic countries (Kajander‐Unkuri et al. 2021; Cabrera and Zabalegui 2021; Löfmark et al. 2015; Sjölin et al. 2019). A joint review of the curriculum and harmonised evaluation could benefit careers, patient safety, and workforce mobility.

It is noteworthy that nearly one in five graduating nursing students have expressed intentions to change careers at an early stage based on the results from this study. Additionally, low scores on self‐assessed clinical competence were associated with an increased likelihood of leaving the nursing profession. This result confirms what other studies found concerning graduates high risk of leaving their careers (Flinkman and Salanterä 2015; Heinen et al. 2013; Flinkman 2014; Flinkman et al. 2010, 2013). Therefore, it is crucial to investigate ways to support student nurses throughout their education and after graduation.

Conclusively, the result of this study provides an overview of the current state of nursing students' self‐assessed clinical competence and perceived need for further training, when about to graduate from general nursing education at bachelor's level. The results can aid educational institutions and healthcare organisations in evaluating, redesigning, and developing nursing education, trainee programs, and continuing education in specialised areas. Developing guidelines for health care leaders and clinical educators to improve and design educational programs can help nursing students and new graduates acquire necessary skills and competencies, keeping them engaged in their profession and reducing the likelihood of leaving it (Forsman et al. 2020; Van Horn and Lewallen 2023; Charette et al. 2020). Additionally, the results are useful to point direction for competence development programs for newly graduated nurses in clinical settings. Additional studies are necessary to explore variations between countries and other background factors that may be related to self‐reported clinical competence for example, the duration of education, clinical experience, and characteristics of completed practical placements during the studies.

### 4.1 Strength and Limitations of the Work

The study was conducted in accordance with the STROBE checklist for cross‐sectional studies (Axley 2008). Several limitations are acknowledged and discussed below. First, data collection occurred during the pandemic, which may have adversely affected the response rate and introduced variations across countries. For instance, it was not possible to provide oral information about the study to students in all participating countries. Moreover, the pandemic may have influenced the findings, potentially increasing the proportion of students expressing intentions to leave the profession. Despite these challenges, the study successfully adopted a Nordic approach, incorporating data from educational institutions across five Nordic countries. However, it is important to note that response rates varied between countries.

When conducting statistical analyses, the insufficient sample size for the use of methods based on the null hypothesis significance testing framework, when comparing countries, was noticed and an estimation approach was selected for those analyses. However, the preferred p‐limit was set at 0.01, to avoid type I errors, but still being aware that, as a consequence, type II errors increase to some extent, because there is a trade‐off between type I and II errors. Thus, due to the possibility of a type II error, even some of the possible true differences may remain non‐significant at the predetermined p‐limit level. High power in a study indicates a greater chance of detecting a true effect, while low power indicates a smaller chance of detecting a true effect. Decreased power does not prohibit the presentation of statistically significant differences, but a methodologically different analysis approach may be preferable, as was also chosen in our study (Dumas‐Mallet et al. 2017). As the data were collected via a research collaboration, the power of this sample was calculated afterwards. The case mean substitution technique was used as an imputation method, as this method for imputation is reported to be adequate for data consisting of self‐reported measures (Fox‐Wasylyshyn and El‐Masri 2005). Only 0.17% of all item values were imputed, which can be seen as a strength. Variables outside the study's scope may influence self‐assessed clinical competence for example, contexts of clinical placements, clinical experience, and length of the education.

When using self‐assessment as a tool to measure clinical competence, it is important to consider the students' individual reflective capacity and consider biases related to self‐evaluation (Bradley et al. 2022; Forsman et al. 2020; Kiekkas et al. 2019; Kajander‐Unkuri et al. 2016). The data pattern shows a correlation between the A scale and the B scale. Eight of the 10 items with the highest mean scores for self‐assessment of clinical competence had the lowest mean scores for the need for further training. Similarly, eight of the items with the highest mean scores for the need for further training were also among the 10 items with the lowest mean scores for self‐assessment of clinical competence. This phenomenon has previously been identified in studies that used the PROFFNurse SAS questionnaire with different samples than the one used in this study (Allvin et al. 2020; Taylor et al. 2020). The pattern suggests that students are providing an honest evaluation of their self‐assessed clinical competence and acknowledging their need for additional training. Based on the findings from this study and previous studies (Allvin et al. 2020; Taylor et al. 2020) the questionnaire could benefit from being further developed by retaining only the A scale for self‐assessment of clinical competence. A shorter questionnaire may increase the response rate in future studies (Mikkonen et al. 2022). The response rate of students who started to answer the questionnaire (*n* = 839) was 29%. However, many students did not complete the survey, resulting in a decreased response rate.

All students were invited to participate via an electronic questionnaire, which requires an active response from the student and may lead to lower response rates. However, the low response rate may have been influenced by the lengthy questionnaire, absence of verbal information, and lack of opportunity to respond in class. This raises the possibility of response bias, as only the most responsible students, who may have the highest grades, may have completed the questionnaire. Therefore, the responses may not be fully representative of all students who are about to graduate from general level nursing education. The version of the questionnaire used (ProffNurse SAS II) has not yet undergone psychometric testing across the Nordic countries, although it has been planned to be conducted in a following study on a larger and more representative sample.

The participant imbalance may limit the generalisability of the results. A sample was taken from higher education institutions in each country, except for Iceland, which was a total sample. It was difficult to predict the variations in the number of students who were in the last semester and would graduate in the different countries. There is also variation in the total number of universities providing nursing education across the countries. There were no significant differences that could be seen to have affected the participation in the data collection protocol among the included countries. The only variation was due to pandemic restrictions, which led to oral information being provided only in Iceland.

### 4.2 Recommendations for Further Research

Future studies regarding Nordic countries should consider focusing on stratification to ensure that roughly equal numbers of participants from all Nordic countries are included, addressing any imbalance and enhancing validity. Sample size calculations should be considered beforehand to ensure efficient power to do comparisons across countries. To increase the response rate and involve even the less active students, shorter questionnaires can be used, oral information can be provided, and students can be invited to answer in class.

## 5 Conclusion

Graduating nursing students from bachelor's level programs in the Nordic countries rate their self‐assessed clinical competence highly in areas essential to nursing from both a caring science and future‐oriented perspective, such as ethical decision‐making, critical thinking, collaboration, and consultation. However, there is a notable gap in clinical competence regarding medication interactions and side effects, and the use of eHealth solutions, underscoring the need for additional training in these areas. Future research may want to compare differences between countries.

Considering the similarities between the Nordic countries and nursing education at a general level, healthcare leaders, clinical teachers, and supervisors of clinical placements should consider the strengths, limitations, and career aspirations of newly qualified nurses when planning and implementing mentoring and professional development programs. Universities should prioritise continuous education and evaluate curricula to enhance the clinical competence of nursing students in critical areas such as pharmacology, and especially in eHealth. By addressing these gaps, the nursing profession will be better equipped to meet current and future demands, ensuring the delivery of high‐quality care in an evolving healthcare sector within the Nordic region.

## Author Contributions

**Lotta Eronen:** conceptualization, methodology, formal analysis, investigation, data curation, writing – original draft, visualization. **Camilla Strandell‐Laine:** conceptualization, methodology, validation, resources, writing – review and editing, supervision. **Sigrid Wangensteen:** conceptualization, methodology, validation, resources, writing – review and editing, supervision. **Auvo Rauhala:** conceptualization, methodology, formal analysis, validation, resources, writing – review and editing, supervision. **Irene Aasen Andersen:** conceptualization, methodology (translation of the (PROFFNurseSAS II, validation of the translation process)), investigation (data collection), resources, writing – review and editing. **Jette Henriksen:** conceptualization, methodology (translation of the (PROFFNurseSAS II, validation of the translation process)), investigation (data collection), resources, writing – review and editing. **Margrét Hrönn Svavarsdóttir:** conceptualization, methodology (translation of the (PROFFNurseSAS II, validation of the translations process)), investigation (data collection), resources, writing – review and editing. **Lisbeth Fagerström:** conceptualization, methodology, validation, resources, writing – review and editing, supervision.

## Ethics Statement

A research permit was obtained from all participating universities, university colleges, or universities of applied sciences by members of the research group in each Nordic country. Data was collected in collaboration with a Nordic research project. The project was approved by the ethical committee of the Norwegian Centre for Research Data (NSD: approval no. 803827). Informed consent was obtained from all participants and by answering the questionnaire, the respondents (graduating nursing students) have given their consent to participate. This study was conducted in accordance with relevant guidelines and regulations. The respondents were informed in writing about the study and when feasible, students were provided with verbal information about the study, considering the restrictions imposed by the COVID‐19 pandemic. The students were also informed about participant anonymity and their right to withdraw from the study at any time, without giving any reason. All methods were implemented in accordance with the EU's General Data Protection Regulation (Regulation 2016/679), and the study adhered consistently to STROBE guidelines.

## Conflicts of Interest

The authors declare no conflicts of interest.

## Data Availability

The datasets generated and/or analysed during the current study are not publicly available due to an ongoing doctoral thesis based on the collected data, including this study as a sub study, but are available from the corresponding author on reasonable request.
